# Growth and reproductive performance responses to post-weaning supplementation of early and normally-weaned Brahman crossbred heifers raised in tropical rangelands

**DOI:** 10.1371/journal.pone.0263786

**Published:** 2022-02-10

**Authors:** Tiago A. C. C. Silva, Simon P. Quigley, Lisa J. Kidd, Stephen T. Anderson, Stuart R. McLennan, Timothy J. Schatz, Kieren D. McCosker, Dennis P. Poppi

**Affiliations:** 1 School of Agriculture and Food Sciences, The University of Queensland, Gatton, Australia; 2 School of Veterinary Science, The University of Queensland, Gatton, Australia; 3 School of Biomedical Sciences, The University of Queensland, St Lucia, Australia; 4 Centre for Animal Science, Queensland Alliance for Agriculture and Food Innovation, The University of Queensland, Dutton Park, Australia; 5 Department of Industry, Tourism and Trade, Darwin, Australia; 6 Department of Industry, Tourism and Trade, Katherine, Australia; Tokat Gaziosmanpasa Universitesi, TURKEY

## Abstract

This study investigated the effect of five post-weaning supplementation strategies and two weaning weight groups on long-term growth, puberty and pregnancy percentage of Brahman crossbred heifers. Early-weaned (118 ± 6 kg liveweight) and normally-weaned (183 ± 6 kg liveweight) heifers were allocated to group pens (n = 4 and n = 5/pen for early- and normally-weaned respectively) and offered one of five levels of post-weaning protein supplementation: 0, 1, 2.5, 5 and 10 g of supplement/kg liveweight.day with *ad libitum* access to a low quality sabi grass (*Urochloa mosambicensis*) hay during the first dry season (169 days) after weaning. After the post-weaning supplementation period, all heifers grazed the same pastures as a single mob until the end of the experiment and were exposed to fertile bulls from January to May 2016. During the first dry season, supplement intake had a positive linear effect on liveweight gain and hip width gain with no difference in the response between weaning groups. Overall, heifers with higher supplement intakes (i.e. 5 and 10 g/kg) had higher hip height gain (*P* < 0.005), hip width gain (*P* < 0.001), body condition score (*P* < 0.001), and concentration of insulin-like growth factor-1 (*P* = 0.001), triiodothyronine (*P* = 0.04) and insulin (*P* = 0.05) in plasma compared to unsupplemented heifers. These changes resulted in thicker proliferative and hypertrophic zones (both *P* = 0.03) of the tuber coxae growth plate, larger diameter of terminal hypertrophic chondrocytes (both *P* = 0.004) at the end of the post-weaning supplementation period when comparing the highest level of supplementation with unsupplemented group. Unsupplemented heifers from both weaning weight groups demonstrated compensatory liveweight gain over the first wet season while evidence of catch-up growth in skeletal dimensions was observed in the second wet season. The main determining factor for pregnancy status of two-year-old Brahman crossbred heifers was pre-mating liveweight (*P* < 0.001), the pre-mating liveweight was in turn affected by post-weaning supplementation (*P* = 0.02) or weaning weight group (*P* < 0.001). This study further demonstrated the positive relationship between premating weight and the occurrence of pregnancy, with an approximate 300 kg pre-mating liveweight required to achieve approximately 80% (67.1–90.3% for a 95% confidence interval) probability of pregnancy in two-year-old Brahman crossbred heifers mated for 4 months.

## Introduction

Decreasing the age at first calving is one management strategy to increase the economic and biological efficiency of a cow-calf production system [1–3]. The age at which beef heifers should be first mated depends upon the economics of management input against returns required for heifers to reach suitable target pre-mating liveweight (LW) by the start of mating, which is dependent on the quality and quantity of available feed and the genetics of the herd [4, 5]. Moriel et al. [6] and Smith and Fordyce [7] suggested that *Bos indicus* crossbred replacement heifers would need to attain approximately 70% of their mature LW by the beginning of the mating season in order to achieve an acceptable pregnancy percentage whereas for *Bos taurus* heifers the recommended pre-mating LW (PM.LW) is usually 60 to 65% [8, 9]. In extensive tropical grazing systems, the nutrition of the cow herd is limited by seasonal variations in the availability and nutritional value of the pastures. In this scenario, early-weaning of calves at the end of the wet-season is commonly adopted as a mean to decrease nutrient requirements and preserve body condition score (BCS) of cows, resulting in an increased re-conception rate of pregnant cows [10–14].

Post-weaning supplementation of early-weaned (EW) heifers has been shown to accelerate growth rates and achieve comparable pregnancy percentage to normally-weaned (NW) Brahman crossbred heifers when mated at one year of age in intensive sub-tropical grazing systems [6]. However, limited information is available to assess the long-term effects of post-weaning supplementation on EW replacement heifers raised in extensive tropical grazing systems, such as northern Australia. In these environments, the average weaning weight (WW) and subsequent LWG during the dry periods of the year is lower, when compared to more intensive grazing systems, such as those described by Moriel et al. [6] and Schubach et al. [15] where > 0.5 kg/day LWG is maintained until mating and WW often exceeding 230 kg. Compensatory growth in LW and catch-up growth in skeletal size are expected to play a role in dictating mature LW and frame size of replacement heifers grazing unimproved pastures in the rangelands of northern Australia. As such, any possible benefit of post-weaning supplementation may be eroded over the subsequent seasons prior to mating. On the other hand, in these environments the practice of early weaning without supplementation is associated with a higher risk of weaner mortality, a longer growing period to overcome the LW deficit relative to un-restricted counterparts and a risk of a permanent reduction of mature skeletal size and lower pregnancy rates at the maiden mating [16, 17].

The main hypothesis of this study is that heifers fed high levels of supplementation would achieve greater puberty and pregnancy percentages when compared to unsupplemented cohorts even after compensatory and catch-up growth partially offsets the differences obtained through supplementation during the first dry season. Unsupplemented EW heifers, were expected to be permanently affected by nutritional restriction and therefore demonstrate lower LWG and skeletal growth rates when compared to NW and supplemented EW counterparts. Therefore, the main goal of this study was to evaluate the impact of different levels of postweaning supplementation in EW and NW Brahman crossbreed heifers on postweaned growth and reproductive performance.

## Materials and methods

### Animal ethics

The procedures realized in this work were in accordance with the Australian Code of Practice for the Care and Use of Animals for Scientific Purposes and were approved by The Charles Darwin University Animal Ethics Committee (A14002).

### Experimental sites and periods

**Fig 1:**
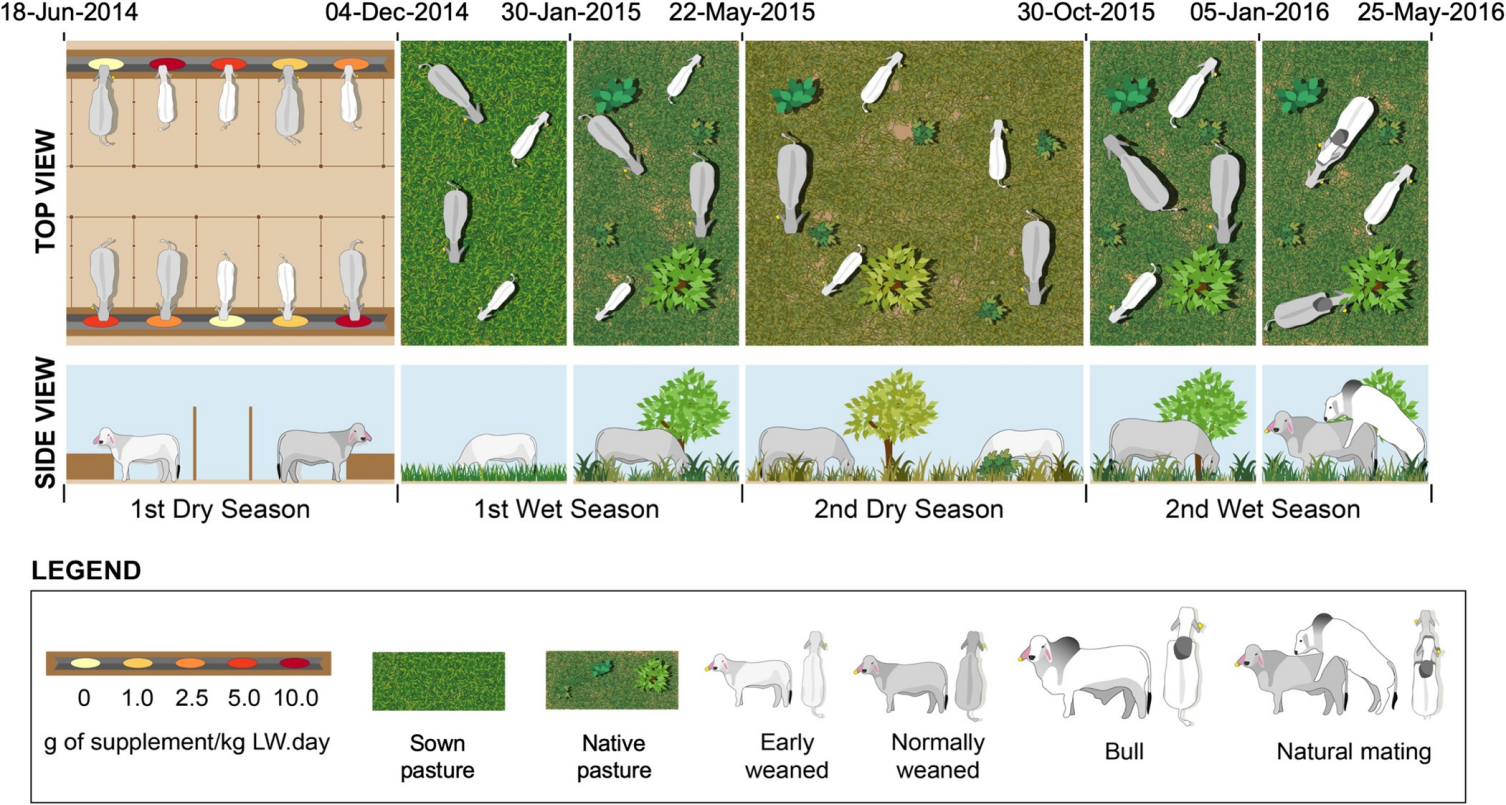
Schematic representation of experimental timeline and animal management during the experiment.

The experiment was conducted over two years in the Northern Territory (NT), Australia as described in Fig 1. The experiment was initiated at the Katherine Research Station (KRS; 14.4737° S, 132.3055° E, Katherine, NT, Australia) where the experimental activities were conducted over the first dry season and the first 57 days of the first wet season, subsequently the experimental animals were transferred to Victoria River Research Station (VRRS; 16°7’S, 130°57’E, Katherine, NT, Australia) for the remainder of the study period. No further experimental manipulations of the animals were performeded at the second site (i.e. VRRS) as indicated in the study desing description as all groups were commingled and managed as one mob. This tranference was necessary due to the fact that the area required to raise these heifers was only available at VRRS whereas the feedlot facilities and improved pastures were only available at KRS. All heifers were transported by truck over a distance of approximately 340km and no major effects of this transportation would be expected to influence the results presented in this work.The experimental periods are described as first dry season (169 days; 18-Jun-2014 to 4-Dec-2014), first wet season (168 days; 5-Dec-2014 to 22-May-2015), second dry season (160 days; 23-May-2015 to 30-Oct-2015) and second wet season (207 days; 31-Oct-2015 to 25-May-2016).

The climate in this region is classified as tropical by the major classification system of Köppen [18], with a short intense wet season followed by a long dry season. Long-term annual rainfall is 971 and 758 mm at KRS and VRRS respectively with 84% of this occurring between December and March each year at both sites [19]. There was higher than normal rainfall over the 2015 wet season, particularly during December at both KRS and VRRS. Otherwise, total annual and monthly distribution of rainfall during the experiment were comparable to the long-term trends at both sites [19]. The three main soil types of VRRS are cracking clays, calcareous red earths and sandy red earths. Soils are generally low in phosphorus (< 5 ppm) and the main native pasture species encountered are *Chrysopogon fallax*, *Iseilema* spp., *Enneapogon* spp., *Heteropogon contortus* and *Sehima nervosum* [20–22].

### Experimental design, animals, diets and feeding

One hundred and thirty-five Brahman crossbred heifers (the heifers had a minimum of 75% Brahman content) were selected for inclusion in the experiment. The heifers were weaned, treated for external parasites (Dectomax [Doramectin 10 mg/L], Pfizer; West Ryde, NSW, Australia), vaccinated against clostridial diseases (5 in 1 Websters, Virbac; Milperra, NSW, Australia) and botulism (Ultravac Botulinum, Pfizer; West Ryde, NSW, Australia) and managed as a single mob with access to 100 g/head.day of copra meal (923 g dry matter [DM], 935 g organic matter [OM], 180 g crude protein [CP], 506 g ash-free neutral detergent fibre [NDF], 4.7 g phosphorus [P] and 0.7 g calcium [Ca]/kg DM) for 30 days (adaptation period). The classification of EW and NW adopted in Northern Australia and in this work is based on the LW of the calf at weaning where calves between 100 and 150 kg LW are classified as EW and over 150 kg LW as NW [14].

The experimental design was a 2 x 5 factorial composed of two weaning LW (WW) and five levels of post-weaning supplementation (Sup) with three pens (replicates) for each treatment. Prior to the experiment, heifers were classified into EW (118 ± 6 kg LW) and NW (183 ± 6 kg LW) on weaning day. Heifers were allocated to pens (n = 4 or n = 5/pen for EW and NW respectively) and blocked on initial LW (i.e. blocks were formed with heifers of similar LW) and pen geographical distribution following the recommendations of Festing [23]. Initial LW was measured 30 days after weaning, at the end of the adaptation period. The five levels of Sup were 0, 1, 2.5, 5 and 10 g supplement/kg LW.day (S0 [unsupplemented], S1, S2.5, S5 and S10 respectively). For the first 72 days of the first dry season, heifers were fed copra meal (Copra) and for the remaining 97 days heifers were fed copra meal and cracked corn (*Zea mays* L.; 909 g DM, 935 g OM, 78 g CP, 152 g NDF, 2.3 g P and 0.1 g Ca/kg DM) in equal portions on an *as fed* basis (Copra:Corn). All heifers had *ad libitum* access to sabi grass (*Urochloa mosambicensis*; 897 g OM, 38 g CP, 612 g NDF, 1.6 g P and 2.8 g Ca/kg DM) hay and a mineral block ([Rumevite: 30% Urea + P; Ridley Agriproducts, Toowoomba, QLD, Australia], 890 g CP, 20 g P and 70 g Ca/kg DM). Hay was chopped to approximately 20 mm in length (NDE 1402; New Direction Equipment, Sioux Falls, SD, USA) prior to feeding and was fed in a separate trough to the supplement.

Supplement allowances for each pen were prepared every 7 days based on total LW of the pen measured every 14 days with allowances adjusted after each weighing. Supplement allowances were offered at 0730 h each day and adjusted volumetrically depending on the previous day’s intake, with residues accumulated in the feed trough over the week. At the end of each week, accumulated supplement residues were collected from the trough, weighed and DM content determined; any supplement not fed was also weighed. Supplement intake per pen was determined as the difference between the total amount of supplement prepared at the start of the week minus the accumulated residues collected from the feed trough at the end of the week plus the amount of supplement not offered.

Sub-samples of supplement offered were collected weekly and bulked every 28 days for subsequent analysis. Hay sub-samples were collected daily and bulked every 28 days. On 20-Aug-2014 and 8-Oct-2014 average hay intake was determined for each pen over seven consecutive days by collecting and weighing the hay refusals at the end of each measurement period, subtracting from the total hay offered and dividing by the number of heifers in each pen. Samples of hay offerings and refusals were collected daily and pooled within collection period and pen. Sub-samples of each period and pen were then utilised for DM determination and chemical analysis.

At the end of the first dry season, all heifers were combined and transferred to a sown pasture at KRS composed of Mekong grass (*Urochloa brizantha* cv. Mekong; 915 g OM, 54 g CP, 528 g NDF, 1.1 g P and 2.4 g Ca/kg DM) and Altum grass (*Panicum altum*; 889 g OM, 60g CP, 627 g NDF, 1.0 g P and 4.1 g Ca/kg DM). Heifers grazed the sown pasture at KRS at 4.8 heifers/ha for 57 days and were offered *ad libitum* access to mineral loose lick supplement (50% salt, 35% Kynophos [21% P, 16% Ca and 1.5% Mg; Brisbane Export Corporation, Brisbane, QLD, Australia] and 15% ammonium-sulphate [Gran-am; Incitec-Pivot, Brisbane, QLD, Australia]). The heifers were then transferred to VRRS (30-Jan-2015) where they remained until the end of the experiment. Stocking rates at VRRS followed the usual regional protocol based on Dyer et al. [24] and during the experimental period averaged 7.5 animal equivalent (AE)/km^2^, where each AE represents a 455 kg steer consuming an estimated 57.1 MJ/day. At VRRS all heifers were managed as a single group with *ad libitum* access to mineral loose lick supplements containing 50% salt, 35% Kynophos and 15% Gran-am in the first and second wet seasons and 50% salt, 10% Kynophos, 25% urea and 15% ammonium-sulphate in the second dry season.

Heifers were exposed to bulls for mating from 05-Jan-2016 to 25-May-2016 (141 days), using only bulls that had successfully passed a standard bull soundness evaluation and semen testing. Heifers were vaccinated against bovine venereal campylobacteriosis (Vibrovax; Pfizer, West Ryde, NSW, Australia) prior to mating.

### Liveweight, body measurements and body condition score

During the first dry season, unfasted LW of heifers was measured prior to feeding every 14 days at the same time on each occasion. Liveweight gain for this period was calculated by regression of change in LW over time. During the subsequent grazing periods, all LW measurements were made after a 15 h feed curfew (i.e. feed restriction) but with free access to water. The LWG for these subsequent periods were calculated as the difference between two subsequent LW measurements divided by the number of days between each measurement. The LW measured at the end of the second dry season was used to generate the relationship between PM.LW and pregnancy status.

Hip height was measured using a tape suspended from a support structure in the crush that was able to move back and forwards in the horizontal direction to adapt for different body lengths. The measurement was taken at the highest point of the sacrum between the tuber coxae bones. Hip height was then calculated by the difference from the distance to the ground and each individual animal measurement. Hip width (HW) was defined as the distance between the most distant points of the tuber coxae on either side of the animal and was measured using an adapted calliper to decrease any possible soft tissue influence on the measurement. Hip height gain (HHG) and hip width gain (HWG) were calculated by regression of each variable over time for the first dry season. For the subsequent periods, both variables were calculated as the difference between two subsequent measures divided by the number of days between measurements. Hip height gain and HWG are presented as mm/100 days. Body condition score was measured at the end of each experimental period using a 1 to 5 scale where 1 is emaciated and 5 is obese [25].

### Blood samples

Four heifers from each pen allocated to S0 and S10 treatments from both WW groups were selected for blood collection throughout the experiment. Blood samples were collected at the start and end of the first dry season, early in the first wet season at KRS and at the end of the first wet season at VRRS. Blood samples were collected from the jugular vein into 10mL lithium heparin coated vacutainers (Becton Dickinson; Franklin Lakes, NJ, USA). After collection the vacutainers were centrifuged at 1700 *g* for 10 min at 4°C and plasma was stored at -20°C until analysis.

### Bone samples and histomorphometry

Within the heifers selected for blood sample collection, another sub-group of heifers (n = 2/pen) was selected for bone biopsies. Biopsies were collected at the end of the first dry season (02-Dec-2014) and early in the first wet season (06-Jan-2015). Before each biopsy, the skin and deeper tissue over the tuber coxae were infiltrated with 35 to 40 mL of lignocaine hydrochloride (20 mg lignocaine hydrochloride/mL; Troy laboratories; Glendenning, NSW, Australia) and left for 5 min for effect. The biopsies were collected from the tuber coxae bone from alternate sides of the same heifer on each occasion. The surgical procedures, and the sample storage, processing and image analysis were as described by Silva et al. [26] with proliferative (PZ) and hypertrophic (HZ) zone heights, number of hypertrophic chondrocytes (HC) per column and diameter of terminal hypertrophic chondrocytes (THC), bone volume (Bv/Tv), trabecular separation (Tb.Sp), trabecular thickness (Tb.Th) and bone surface (BS) determined. The surgical procedure adopted in this study is novel but it was also performed in a similar study conducted by our lab [26] and offers the advantage of sampling the growth plate tissue without the necessity of euthanizing the experimental animals as is commonly adopted in this research field [27, 28].

### Ovary scanning and pregnancy diagnosis

All heifers heavier than 250 kg LW at the end of the second dry season were ovary scanned using an ultrasound (HS-2200, Honda Electric, Inc.; Toyohashi, Japan) with a 7.5 MHz linear interoperative 38 mm probe by trans-rectal examination and classified according to the presence of the corpus luteum (CL) or not. The process was repeated 10 days later in order to detect possible heifers that were cycling but did not have a developed CL at the first scanning. All ovary scanning and pregnancy testing were conducted by the same experienced operator. All heifers with a CL on at least one of these occasions were considered to have reached puberty. At the end of the second wet season all heifers were pregnancy tested by rectal palpation and ultrasonography. The pregnancy diagnosis was conducted by a single experienced operator. Both variables (i.e. pubertal and pregnancy status) were recorded as a binomial outcome and were coded as “1” for pubertal or pregnant and “0” for non-pubertal or non-pregnant.

### Laboratory analysis

Sub-samples of feed offered and feed residues were dried in duplicate to a constant weight at 65°C for DM determination. Samples were then ground through a 1 mm screen (Retsch ZM 200; Haan, Germany), dried for 24 h at 105°C to determine residual DM content and then combusted in an electric muffle furnace (Modutemp Pty. Ltd.; Perth, WA, Australia) for 8 h at 550°C to determine ash and OM content. The N content of feed offered was measured by the Kjeldahl method using an auto-digestor (Tecator 2520, FOSS; Hillerød, Denmark) and a N analyser (Kjeltec 8400, FOSS; Hillerød, Denmark) following the manufacturers guidelines. Crude protein content was calculated using the conversion factor 6.25 x N. The content of ash-free NDF in feeds offered were measured following the procedure developed by Van Soest et al. [29] using a fibre analyzer (A200, Ankom; Macedon, NY, USA) and described in detail by Silva et al. [26]. The ash content was determined, and ash-free NDF content was calculated. The mineral content of feeds offered was determined on an ICP-OES spectrometer (Optima 7300 DV, PerkinElmer; Waltham, MA, USA) after a nitric-perchloric acid digestion. The nitric-perchloric solution was prepared using 6 mL of nitric acid, 2 mL of perchloric acid and 12 mL of reversed osmosis water.

The concentration of nonesterified fatty acids (NEFA), Ca, inorganic phosphorus (PiP), urea (PUN) and total protein in plasma were measured using an Olympus AU400 auto-analyser (Beckman Coulter Diagnostic Systems Division, Melville, NY, USA). The concentration of insulin and IGF-1 in the plasma were determined by immunoradiometric assays (DIAsource INS-IRMA Kit, DIAsource; Louvain-la-Neuve, Belgium; A15729, Immunotech, Beckman Coulter; Prague, Czech Republic). The inter- and intra-assay coefficients of variation were 5.4 and 7.8%, and 9.5 and 7.7%, for insulin and IGF-1 respectively. Total triiodothyronine was analysed using a radioimmunoassay kit (IM1699, Immunotech, Beckman Coulter). The inter- and intra-assay coefficients of variation for this analysis were 7.8 and 3.6%. Leptin was analysed using a radioimmunoassay kit (XL-85K, Millipore Corporation; St Charles, MO, USA). The inter- and intra-assay coefficients of variation for this analysis were 6.4 and 5.9%. The concentration of the bone markers cross-linked C-terminal telopeptide of type I collagen (CTX-1) and bone-specific alkaline phosphatase (BALP) in the plasma were determined by enzyme immunoassays using commercially available kits (Immunodiagnostic Systems Ltd; Boldon, UK and MicroVue BAP 8012, Quidel; San Diego, CA, USA respectively) on a plate reader spectrophotometer (Sunrise Absorbance Microplate Reader, Tecan; Pheonix, CA, USA) with XFluor software (Tecan; Pheonix, CA, USA). The inter- and intra-assay coefficient of variation were 6.2 and 3.6% for a quality control of 53.9 U/L for BALP and 6.3 and 8.2% for a quality control of 2.4 ng/mL for CTX-1.

### Statistical analysis

All statistical analyses were performed using R statistical software (version 3.6.1) [30]. To screen explanatory variables on their ability to satisfy the general linear model assumptions in their raw form the normality and homoscedasticity was assessed by visual inspection. For all continuous outcome variables, such as LWG, HHG and HWG, the pen was used as the experimental unit. Puberty and Pregnancy were specified as binary outcome variables and were analysed by specifying the animal as the experimental unit with adjustment for the effects of pen by specifying as a random effect. A weighing factor was also included in the model to account for the different number of heifers in EW and NW pens.

To assess the effect of supplement intake and WW on LWG, HHG and HWG during the first dry season data were subjected to a regression analysis. The initial models included the quadratic effect of supplement intake and the interaction between Sup and WW and were removed when not significant (*P* < 0.05). Final models were selected based on the Akaike information criterion [31] and parsimony [32]. Hay, supplement and total intake, LWG, HHG and HWG, bone histomorphometric parameters, and the concentration of metabolites, hormones and bone markers in the plasma were analysed using the linear mixed effects model procedure of the package “nlme” [33]. Weaning weight, Sup and their interaction were included in the model as fixed effects and pen within block as a random factor. The effect of WW, Sup and their interaction on pregnancy was assessed using a generalized linear model with binomial variance and a logit “link”. The *P*-values for main factors were generated using the chi-square test. A similar model using the PM.LW recorded at the end of the second dry season and WW group as an explanatory variable was used to investigate the relationship between LW and occurrence of pregnancy. The confidence interval for the model predictions was calculated on the linear scale of the predictor (i.e. PM.LW) and then transformed applying the inverse link function extracted from the model in order to represent the same scale of the response variable (i.e. pregnancy). Graphs were generated using R package “ggplot2” [34].

## Results

### The effect of supplement intake and weaning weight group on liveweight gain, hip height gain and hip width gain

There was a significant positive association between supplement intake and all variables measured (Table 1). There was no difference in the LWG and HWG responses for EW and NW heifers but there was a significant interaction between WW and supplement intake on the HHG response. Early weaned heifers showed a greater increase in HHG with increasing supplement intake compared to NW heifers.

**Table 1:** The effect of supplement intake (X)^1^ and weaning weight on liveweight gain (LWG), hip height gain (HHG) and hip width gain (HWG) of early (EW) and normally (NW) weaned^2^ crossbred Brahman heifers.

Y	Weaning weight	Response equation	R^2^	RSE^3^
LWG	EW, NW^4^	Y = 0.13 + 0.054X	0.80	0.05
HWG	EW, NW	Y = 8.99 + 3.030X	0.61	4.94
HHG	EW	Y = 53.8 + 4.360X	0.75	4.59
	NW	Y = 51.2 + 2.590X	0.75	4.59

^1^ Supplement was composed of Copra meal for the initial 72 days and Copra:Corn mixture for the remaining 97 days of the first dry season.

^2^ The experimental design is a 2 x 5 factorial including two weaning weight groups (i.e. EW and NW) and five increasing levels of supplementation. Each treatment group combination was replicated in three group pens.The pen average of each parameter was utilized in this analysis.

^3^ Residual standard error (RSE).

^4^ A single regression equation was generated for both weaning groups (i.e. EW and NW) when the weaning weight group effect was not significant.

### Hay, supplement and total dry matter intake during 1st dry season

During the first dry season, hay and supplement intake were significantly (*P* < 0.001) affected by Sup but not WW or its interaction (*P* > 0.05; Table 2). Total dry matter intake was not affected by any of the main treatments or its interaction (*P* > 0.05; Table 2). The maximum Copra intake was 3.9 and 3.3 g DM/kg LW.day for NW and EW heifers respectively. Due to the low voluntary intake of the Copra over the first 72 days of the first dry season, Copra:Corn mix was used as the supplement for the remainder of the first dry season in an attempt to increase supplement and ME intake. Thereafter, supplement intake increased to 7.2 and 6.6 g DM/kg LW.day when offered at S10 for NW and EW groups respectively. Increasing supplement intake led to a significant substitution effect and reductions in hay intake leading to overall similar total dry matter intake across all treatment groups. Overall, supplement intake was lower than expected at the highest supplement allowances but still generated a divergence in LW and HH growth paths during the first dry season.

**Table 2:** Hay (HI), Supplement (SI) and total (TI) intake (g DM/kg LW.day), Liveweight gain (LWG; kg/day), hip height gain (HHG; mm/100 days), hip width gain (HWG, mm/100 days) and body condition score (BCS) of early (EW) and normally (NW) weaned^1^ crossbred Brahman heifers fed different levels of post-weaning supplementation^2^ over the first dry season^3^^,^^4^.

Period	Parameter	Supplementation treatment (Sup)					Weaning weight (WW)		SEM	*P*-value		
		S0	S1	S2.5	S5	S10	EW	NW		Sup^1^	WW^1^	Sup x WW
1^st^ Dry	HI	22.4 a	21.9 a	19.8 ab	17.2 b	16.2 b	20.4	18.7	0.90	<0.001	0.08	0.61
	SI	0 e	0.9 d	2.3 c	4.0 b	5.6 a	2.5	2.6	0.13	<0.001	0.45	0.20
	TI	22.4	22.8	22.1	21.1	21.9	22.7	21.4	1.22	0.73	0.09	0.58
	LWG	0.11 c	0.18 c	0.30 b	0.39 ab	0.42 a	0.26	0.303	0.04	<0.001	0.09	0.18
	HHG	54 c	58 bc	65 ab	70 a	73 a	67	61	3.8	<0.005	<0.01	0.68
	HWG	9.9 b	10 b	17 ab	23 a	24 a	16	17	1.9	<0.001	0.54	0.37
	BCS^5^	2.8 c	3.0 bc	3.1 b	3.4 a	3.5 a	3.0	3.8	0.08	<0.001	<0.001	0.12
1^st^ Wet	LWG	0.41 a	0.41 a	0.39 a	0.36 ab	0.29 b	0.37	0.37	0.035	<0.005	0.86	0.41
	HHG	27	29	30	29	26	34	22	3.5	0.35	<0.001	0.56
	HWG	26	30	29	29	23	29	26	1.7	0.09	0.15	0.58
	BCS	2.9	3.0	3.0	3.0	3.1	2.9	3.1	0.1	0.13	<0.001	0.63
2^nd^ Dry	LWG	0.02	-0.08	-0.03	-0.02	-0.01	0.01	-0.03	0.03	0.16	<0.005	0.77
	HHG	27	29	25	28	23	27	26	4.2	0.48	0.43	0.62
	HWG	19	19	16	19	22	19	19	1.2	0.06	0.46	0.74
	BCS	2.7	2.7	2.7	2.8	2.6	2.6	2.8	0.09	0.19	<0.001	0.72
2^nd^ Wet	LWG	0.49	0.52	0.53	0.50	0.48	0.50	0.51	0.030	0.17	0.33	0.61
	HHG	27 a	21 ab	25 ab	19 b	23 ab	29	17	2.5	<0.01	<0.001	0.82
	HWG	15 ab	18 a	15 ab	12 b	12 b	17	13	1.6	0.03	<0.01	0.65
	BCS	3.0	3.0	3.0	3.0	3.1	3.0	3.1	0.05	0.37	<0.005	0.06

^1,2^ The experimental design is a 2 x 5 factorial including two weaning weight groups (i.e. EW and NW) and five increasing levels of supplementation. Each treatment group combination was replicated in three group pens.The pen average of each parameter was utilized in this analysis.

^3^ Data are least squares means with standard error of the mean (SEM).

^4^ Treatment means within a row with different superscript letters are significantly different (*P* < 0.05).

^5^ BCS recorded at the end of each period using a 1–5 scale (refer to materials and methods for further details).

### Liveweight, hip height and hip width gain, and body condition score

Growth rate of all parameters were affected by Sup during the first dry season (Table 2). Whilst there was no difference in LWG between EW and NW heifers, HHG was higher in EW heifers compared with NW heifers and EW were lower in BCS at the end of the period (*P* < 0.001). Overall, no significant (*P* > 0.05) interactions were found between Sup and WW for growth parameters assessed throughout the experiment.

Heifers with higher supplement intake during the first dry season gained less LW during the following wet season (Table 2). A significant negative linear relationship existed between LWG during first dry season and LWG during the first wet season (Y = 0.44296–0.2649X; R^2^ = 0.31, *P* = 0.004) where for every 0.1 kg/day increase in LWG during the first dry season led to a decrease of 0.026 kg/day in LWG during the subsequent wet season, within the range of 0 to 0.5 kg LWG/day measured during both periods. At the end of the first wet season, heifers offered the S0 treatment post-weaning had recovered 44% of the LW deficit that existed at the end of the first dry season compared to heifers offered the S10 treatment. Liveweight was the only parameter that showed any degree of compensation during this period. Overall, there was a greater HHG by EW heifers compared to NW (34 *vs* 22 mm/100 days; *P* < 0.0001) during the first wet season.

Heifers maintained LW over the second dry season regardless of Sup (*P* = 0.16) during the first dry season. Early weaned heifers had a statistically significant (*P* < 0.01) but biologically irrelevant increase in LWG during this period when compared to NW (0.017 *vs* -0.032 kg/day). At the end of the second dry season, BCS of NW heifers was higher than EW heifers despite the higher LWG achieved by the latter group during the period. During the second wet season, heifers fed higher supplement levels during the first dry season (S5) had lower HHG (*P* < 0.01) and HWG (*P* = 0.03) growth rates when compared to treatments with low (S1) or no supplement (S0) intake during the first dry season. Early weaned heifers also had higher HHG (29 *vs* 17 mm/100 days, *P* < 0.01) and HWG (17 *vs* 13 mm/100 days; *P* < 0.05) than NW heifers during the second wet season but no differences in LWG were observed (0.50 *vs* 0.51 kg/day; *P* = 0.33). The skeletal growth rate (i.e. HHG and HWG) of EW heifers was, for most seasons, greater than NW heifers, however the EW heifers remained smaller and lighter than NW heifers at the end of the experimental period.

### Plasma metabolites, hormones and bone metabolism markers

No significant interaction between Sup (only S0 and S10 were analysed) and WW groups were observed for the concentration of metabolites, hormones and bone markers in the plasma at any collection date (Table 3). The concentration of IGF-1 was higher in the plasma of NW heifers compared with EW heifers prior to commencement of the experiment (i.e. start of the first dry season; *P* = 0.01) with no differences in the concentration of metabolites, other hormones or bone markers measured in plasma at this point in time. The concentration of PUN, insulin, IGF-1 and T3 in the plasma was higher in supplemented compared to unsupplemented heifers (*P* < 0.05) at the end of the first dry season. There was a tendency (*P* = 0.056) for a higher BALP concentration in plasma of supplemented heifers while the concentration of leptin and CTX-1 were unaffected (*P* > 0.05) by supplement intake. There was no effect (*P* > 0.05) of WW on the concentration of metabolites, hormones and bone markers in the plasma during the supplementation period.

**Table 3:** The concentration of calcium (Ca; mmol/L), phosphorus (P; mmol/L), total protein (mg/L), plasma urea-N (PUN; mmol/L), non-esterified fatty-acids (NEFA; mEq/L), insulin (IU/mL), insulin-like growth factor-1 (IGF-1; ng/mL), triiodothyronine (T3; nmol/L), leptin (ng/mL), bone-specific alkaline phosphatase (BAP; U/L) and C-terminal telopeptides of type I collagen (CTX-1; ng/mL) in the plasma of early (EW) and normally (NW) weaned crossbred Brahman heifers fed two different supplement treatments^1^ during the first dry season^2^^,^^3^.

Period	Parameter	EW		NW		SEM	*P*-value		
		S0	S10	S0	S10		Sup^1^	WW^1^	Sup x WW
Start 1^st^ Dry	Ca	1.7	1.7	2.0	1.9	0.1	0.97	0.09	0.54
	P	2.3	2.3	2.5	2.4	0.1	0.84	0.30	0.66
	Total protein	63	64	67	64	2.8	0.95	0.40	0.60
	PUN	2.6	2.9	2.2	2.3	0.2	0.54	0.10	0.75
	NEFA	0.5	0.5	0.5	0.4	0.1	0.60	0.98	0.67
	Insulin	7.1	8.6	8.8	9.6	1.1	0.19	0.13	0.69
	IGF-1	5.8	11.2	25.7	24.5	5.2	0.69	0.01	0.55
	T3	0.74	1.0	1.1	0.95	0.1	0.86	0.29	0.17
	Leptin	5.4	6.8	6.4	7.0	1.1	0.36	0.57	0.68
End 1^st^ Dry	Ca	1.6	1.8	1.8	1.9	0.1	0.07	0.11	0.98
	P	2.0	2.3	2.3	2.2	0.07	0.23	0.20	0.11
	Total protein	58	60	60	62	1.6	0.24	0.32	0.67
	PUN	1.1	1.4	1.2	1.9	0.1	0.02	0.08	0.21
	NEFA	0.3	0.3	0.4	0.4	0.07	0.85	0.32	0.98
	Insulin	7.3	9.8	8.6	15.9	1.7	0.05	0.19	0.56
	IGF-1	9.2	53.7	18.3	100.8	19.3	0.001	0.14	0.66
	T3	1.0	1.8	0.9	1.48	0.2	0.04	0.33	0.84
	Leptin	5.5	6.2	4.9	4.7	0.7	0.94	0.22	0.56
	BALP	35	46	25	35	5.0	0.05	0.08	0.99
	CTX-1	1.7	1.8	1.8	1.3	0.2	0.21	0.17	0.13
Early 1^st^ Wet	Ca	2.1	2.2	2.3	2.3	0.08	0.54	0.17	0.80
	P	2.2	2.2	2.6	2.3	0.1	0.14	0.03	0.18
	Total protein	69	68	72	73	1.6	0.83	0.01	0.37
	PUN	2.2	1.9	2.2	2.5	0.1	0.95	0.03	0.07
	NEFA	0.48	0.35	0.55	0.50	0.03	0.03	0.02	0.30
	Insulin	8.8	7.9	8.3	9.6	1.3	0.68	0.41	0.31
	IGF-1	12.5	32.9	34.4	39.8	7.7	0.03	0.04	0.12
	T3	0.95	0.97	0.94	0.78	0.08	0.29	0.20	0.28
	Leptin	7.0	5.4	4.3	6.4	1.5	0.87	0.52	0.21
	BALP	28	31	42	31	4.5	0.47	0.07	0.10
	CTX-1	1.9	1.1	1.6	1.9	0.2	0.16	0.25	0.06
End 1^st^ Wet	Ca	2.5	2.5	2.4	2.3	0.06	0.63	0.04	0.44
	P	1.3	1.3	1.6	1.2	0.1	0.18	0.63	0.15
	Total protein	75	75	74	76	1.0	0.35	0.63	0.21
	PUN	7.8	7.3	6.9	6.9	0.3	0.53	0.07	0.45
	NEFA	1.4	1.0	1.1	1.4	0.08	0.85	0.43	0.06
	Insulin	7.6	10.2	10.3	8.5	1.3	0.78	0.59	0.14
	IGF-1	25.7	48.2	54.7	66.7	14.0	0.01	0.005	0.23
	T3	1.8	1.8	2.0	1.5	0.2	0.38	0.75	0.37
	Leptin	11.1	9.8	12.3	12.3	1.5	0.53	0.30	0.73

^1^ The experimental design is a 2 x 5 factorial including two weaning weight groups (i.e. EW and NW) and five increasing levels of supplementation. Each treatment group combination was replicated in three group pens.This analysis only included S0 and S10 treatments. The analysis was conducted with the pen average for each parameter.

^2^ Data are least squares means with standard error of the mean (SEM).

^3^ Start and end of first dry and start and end of first wet refer to the following collection dates: 18-Jun-2014, 02-Dec-2014, 06-Jan-2015 and 22-May-2015.

Early in the first wet season (i.e. 33 days after start of wet season) heifers fed treatment S10 during the previous dry season had higher IGF-1 (*P* = 0.03) and lower NEFA (*P* = 0.03) concentration in the plasma compared to S0 heifers (Table 3). The concentration of IGF-1, P, total protein, PUN and NEFA were higher (*P* < 0.05) in the plasma of NW compared to EW heifers early in the first wet season. However, by the end of the first wet season the effects of WW or supplementation were only apparent on the concentration of IGF-1 and Ca in the plasma of heifers.

### Growth plate and trabecular bone histology

Heifers offered the S10 treatment during the first dry season had thicker PZ and HZ and greater HC diameter than unsupplemented heifers (*P* < 0.05; Table 4). There was a significant (*P* = 0.01) interaction effect of Sup and WW groups on the thickness of the PZ of the growth plate. The PZ was significantly thicker in EW heifers offered the S10 treatment compared to unsupplemented heifers with no Sup effect on NW heifers.

**Table 4:** Height of proliferative (PZ; μm) and hypertrophic zone (HZ; μm), number of hypertrophic chondrocytes (n of HC) per column (n/column), diameter terminal hypertrophic chondrocytes (THC; μm), bone volume (Bv/Tv; %), trabecular separation (Tb.Th; μm), trabecular thickness (Th.Sp; μm) and bone surface area (BS; mm^2^) of early (EW) and normally (NW) weaned crossbred Brahman heifers fed two different supplement treatments^1^ during the first dry season^2^^,^^3^.

Period	Parameter	EW		NW		SEM	*P*-value		
		S0	S10	S0	S10		Sup^1^	WW^1^	Sup x WW
End 1^st^ Dry	PZ	184 b	221 a	221 a	218 a	8.4	0.03	0.03	0.01
	HZ	180	220	195	207	9.8	0.03	0.89	0.19
	n of HC	8.3	9.0	8.8	8.9	0.3	0.27	0.62	0.36
	THC	26.3	29.1	24.4	28.2	1.2	0.004	0.11	0.54
	Bv/Tv	20.2	20.9	21.2	21.1	0.1	0.83	0.70	0.78
	Tb.Th	149	158	165	149	5.7	0.62	0.44	0.05
	Tb.Sp	561	540	609	511	42	0.23	0.76	0.41
	BS	41	44	43	43	2.1	0.74	0.90	0.75
Early 1^st^ Wet	PZ	195	214	225	208	8.3	0.90	0.14	0.05
	HZ	194	221	210	191	9.4	0.68	0.49	0.05
	n of HC	8.9	8.2	7.7	7.6	0.5	0.15	0.56	0.61
	THC	25.0	26.6	25.6	24.9	0.8	0.52	0.42	0.12
	Bv/Tv	15.8	19.0	18.3	17.7	0.8	0.19	0.53	0.07
	Tb.Th	129	129	145	137	6.4	0.53	0.11	0.56
	Tb.Sp	644	574	644	524	41.7	0.09	0.78	0.39
	BS	32	39	38	37	1.8	0.21	0.44	0.07

^1^ The experimental design is a 2 x 5 factorial including two weaning weight groups (i.e. EW and NW) and five increasing levels of supplementation. Each treatment group combination was replicated in three group pens.This analysis only included S0 and S10 treatments. The analysis was conducted with the pen average for each parameter.

^2^ Data are least squares means with standard error of the mean (SEM).

^3^ End of first dry and early in the first wet refer to the following collection dates: 02-Dec-2014 and 06-Jan-2015.

No significant effects of Sup, WW or their interaction were observed on histomorphometric assessments of trabecular bone during supplementation or early in the first wet season. None of the morphological differences previously observed at the growth plate were evident early in the first wet season (i.e. after 33 days of grazing).

### Reproduction

No significant interaction (*P* > 0.05) between Sup treatments and WW groups were found for either pregnancy or puberty (Table 5). At the end of the second dry season 33% (SEM = ± 6.0%) of the NW were classified as pubertal, compared to none for EW heifers (*P* < 0.001). Heifers with the highest supplement intake (i.e. S10) over the first dry season had a significantly (*P* = 0.04) higher pubertal percentage than unsupplemented heifers (i.e. S0).

**Table 5:** Puberty and pregnancy percentage of early (EW) and normally (NW) weaned Brahman crossbred heifers fed different post-weaning supplementation treatments during the first dry season^1^^,^^2^^,^^3^.

Parameter	Weaning weight (WW)	Supplementation treatment (Sup)					SEM	*P-*value		
		S0	S1	S2.5	S5	S10		Sup^1^	WW^1^	Sup x WW
LW at weaning, kg	EW	127.1	130.9	129.6	127.2	127.4	6.5	0.25	<0.001	0.96
	NW	191.8	194.3	192.5	190.7	192.3				
	P WW	159.4	162.6	161.0	158.9	159.8				
Pubertal^4^^,^^5^, %	EW	0.0	0.0	0.0	0.0	0.0	12.0	0.04	<0.001	0.99
	NW	13.3	43.3	20.0	40.0	60.0				
	P WW	6.6 a	21.6 ab	10.0 ab	20.0 ab	30.0 b				
Pre-mating LW ^5^, kg	EW	206.5	210.0	223.2	228.0	224.6	10.2	0.02	<0.001	0.88
	NW	258.6	271.3	270.9	280.9	282.4				
	P WW	232.6 a	240.6 ab	247.0 ab	254.5 ab	253.5 b				
Pregnancy ^4^, %	EW	25.0	45.5	25.0	36.4	16.7	14.5	0.33	<0.001	0.40
	NW	53.3	71.4	46.7	86.7	80.0				
	P WW	39.1	59.4	35.8	62.7	48.3				

^1^ The experimental design is a 2 x 5 factorial including two weaning weight groups (i.e. EW and NW) and five increasing levels of supplementation. Each treatment group combination was replicated in three group pens.The pen average of each parameter was utilized in this analysis.

^2^ Data are least squares means with standard error of the mean (SEM).

^3^ Liveweight (LW), pooled means for WW groups (P WW).

^4^ Pubertal status was determined by ovary scanning at the end of the 2^nd^ dry season (30-Oct-2015) and pregnancy percentage was determined by rectal palpation at the end of the 2^nd^ wet season (25-May-2016).

^5^ Different letters within a row denote a significant difference (*P* < 0.05) for supplementation treatment effect (P WW).

There was a significant effect of WW group (*P* < 0.001) but not Sup (*P* > 0.05) on the percentage of heifers pregnant. The overall likelihood of pregnancy predicted by the final model for NW and EW, when expressed as a percentage,was 69.7% (SEM = ± 5.8%) and 28.7% (SEM = ± 6.1%) for NW and EW heifers, respectively. Normally weaned heifers fed S2.5 had a lower puberty percentage and likelihood of pregnancy than what would be expected based on the average PM.LW (Table 4). This group (i.e. NW fed S2.5) also presented the highest variation (SEM = ± 14 kg) in PM.LW when compared to the other treatment groups (all SEM < ± 8.5 kg).

**Fig 2:**
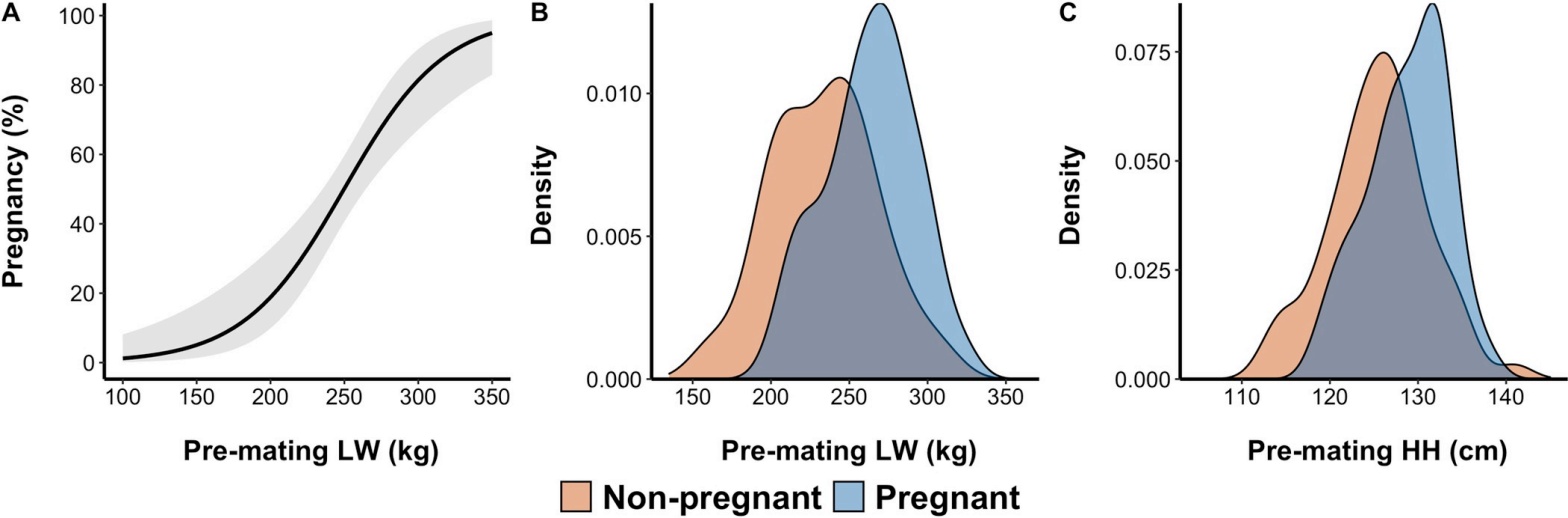
Effect of pre-mating liveweight (LW) on the likelihood of pregnancy, expressed as a percentage, (A) and kernel density estimation plots of pre-mating LW (B) and pre-mating hip height (HH; C) of non-pregnant and pregnant crossbred Brahman heifers. Weaning weight group and its interaction with pre-mating LW was initially included in the model describing the effect of pre-mating LW on pregnancy percentage and since it was not significant (*P* = 0.71 and *P* = 0.90 respectively) a single model was created using data from early and normally weaned heifers. The model represented in A is described by Eqs 1 and 2 and grey shaded area represents the 95% confidence interval around the fit. Pre-mating LW and HH were recorded on 30-Oct-2015 and natural mating occurred between 05-Jan-2016 to 25-May-2016.

After adjusting for the effects of clusting at the pen level, pre-mating LW recorded at the end of Oct-2015 was strongly associated with the likelihood of pregnancy (*P* < 0.001) (Fig 2A).

The initial model also included WW group, Sup and their interaction with PM.LW which were not significant and therefore removed from the final model resulting in the following equations:

$$Pregnancy\;(\%)=\frac{e^{\theta }}{\left(1+e^{\theta }\right)}$$ Eq 1

θ=−7.3353+0.0293PM.LW Eq 2

Where pregnancy percentage is the estimate of the probability of pregnancy (Eq 1) and PM.LW (Eq 2) is the feed curfew LW recorded on at the end of second dry season. On average, pregnant heifers were 30 kg heavier (263.3 *vs* 233.7 kg; Fig 2B) and 32 mm (1287 *vs*. 1255 mm; Fig 2C) taller than non-pregnant heifers at the time of PM.LW.

## Discussion

This experiment examined the effect of Sup and WW on long-term growth, puberty and pregnancy of Brahman crossbred heifers raised in tropical rangelands. The results presented demonstrated that post-weaning supplementation above 2.5 g supplement/kg LW.day increased LWG and skeletal growth and this was associated with higher plasma concentration of IGF-1, T3 and insulin as well as thicker proliferative and hypertrophic zones and wider diameter of terminal hypertrophic chondrocytes during the post-weaning dry season. The effect of supplementation on the assessed growth and reproductive parameters was in general similar between EW and NW heifers during supplementation and over the subsequent experimental periods. However, the regression analysis showed that increasing supplement intake resulted in a higher increase in HHG for EW than NW heifers. Unsupplemented heifers demonstrated compensatory growth in LW during the first wet season while evidence of skeletal catch-up growth was observed over the second wet season. There was no indication that the absence of post-weaning supplementation to EW heifers would have caused any detrimental impact on their long-term growth or puberty and pregnancy percentage. Post-weaning supplementation led to greater PM.LW and pubertal percentage. However, contrary to our initial hypothesis, it did not lead to significant increases in pregnancy percentage as unsupplemented heifers were able to reach adequate LW by the end of mating as two-year-old to achieve a similar pregnancy rate to heifers that had been supplemented over the post weaning dry season. However, the percentage of NW heifers offered 5 or 10 g of supplement/kg LW.day that were confirmed pregnant was numerically greater (83.4 *vs* 57.1%) than heifers that received low or no supplementation. Normally-weaned heifers had a significantly higher pregnancy percentage than EW heifers independent of post-weaning supplement intake over the first dry season. Overall, the parameter that explained the greatest variation for occurrence of pregnancy was PM.LW, which was, in general, greater for the NW compared with EW heifers. This is consistent with previous studies which have found that the most important factor affecting pregnancy rates of maiden heifers is PM.LW [35, 36]. This outlines the long-term consequence of first dry season supplementation and WW on pregnancy percentage, a practical objective of this experiment. However, the underlying reasons behind the long-term consequence of WW and supplement intake during the first dry season are explored further below in relation to LW, BCS and skeletal frame size.

### Copra meal as a supplement for weaned calves

The maximum voluntary intake of copra meal was lower than expected, and previously reported in the literature, when fed with and without the addition of cracked corn (3.5 and 6.9 g DM/kg LW.day for Copra and Copra:Corn respectively) [37, 38]. The reasons for the lower than expected supplement intake in the current experiment are unknown. During the experiment no obvious problems with the copra meal were noticed. Despite being lower than expected, the range of supplement intake achieved in the first dry season resulted in differences in LW and HH which was the main objective of this experimental design.

A positive linear relationship between supplement intake and LWG, HHG and HWG was observed. The interaction between supplement intake and WW was significant for HHG, showing a greater increment in HHG for increasing supplement intake in EW than NW heifers. A linear relationship between supplement intake and LWG, HHG and HWG was not expected. McLennan [37] investigated the effect of increasing copra meal supplement intake on LWG of Brahman x Angus steers and observed a quadratic response where the highest LWG (~0.6 kg/day) was observed at approximately 8 g of supplement/kg LW. It is likely that the polynomial degree (i.e. linear or quadratic) of the relationships in the present experiment were affected by the lower than expected voluntary intake of supplement. It is well described in the literature that the response for increasing intake of protein supplementation to growing cattle fed low quality forages is steeper at lower supplement intake followed by a more gradual increase at higher supplement intakes [39–41].

The different response in HHG for EW and NW over increasing supplement intake may be at least partially explained by the different growth plate maturity in the tuber coxae of the two groups. It has been previously demonstrated that for a given bone maturity, nutrition is a major determinant of bone growth rate in cattle, where approximately 82% of the variation in HHG was explained by CP intake [26]. However, the effect of morphological and physiological changes undertaken by the growth plate itself during its growth history would be expected to be the main factor limiting the bone elongation rate of cattle in a given diet [42]. In general terms, it implies that EW heifers with a less mature growth plate would have a greater bone elongation potential and therefore a greater response in HHG when offered high quality nutrition when compared to NW heifers.

### Effect of first dry season supplementation and weaning weight on subsequent liveweight gain

It is well established that following a period of nutritional restriction, animals can undergo compensatory growth in LW if offered *ad libitum* access to feed with high nutritional value [43–45]. These observations are consistent with the results of the present study. Unsupplemented heifers showed a greater LWG increase over the first wet season than supplemented heifers although in this study DM intake was not measured during the grazing phase.

Approximately 53% (average of S0, S1 and S2.5 treatments including both WW groups) of the initial LW difference achieved by feeding supplements during the first dry was compensated in the subsequent wet season. By the start of the second wet season, this LW difference was further increased to 66%, which was maintained until the end of the experiment. A 100% compensation would mean that any possible greater LWG achieved through supplementation during the first dry season would have been eroded after the second wet season. The negative linear relationship between first dry and first wet season LWG was independent of WW and only existed for LW but not the skeletal measurements. Overall, there was no evidence of any long-term negative effect on growth of EW heifers when fed only a mineral urea supplement (i.e. S0) during the first dry season. However, EW heifers entered the mating period on average at a much lower LW (218.5 *vs* 272.8 kg; *P* < 0.001) and BCS (2.6 *vs* 2.8 units; *P* < 0.001) than NW heifers, a lower percentage were cycling as determined by ovarian ultrasound scanning (0 *vs* 35%; *P* < 0.001) and this was associated with a significantly lower pregnancy percentage compared to NW heifers (30.5 *vs* 67.6%; *P* < 0.001). Thus, the difference in WW does not impair the growth of the animal, but a heifer still needs to reach a threshold target LW and BCS to have a high probability of pregnancy. Therefore, in an extensive tropical pasture rangeland production system extra inputs are required over the growth path between weaning and mating if an EW heifer is to achieve high (i.e. > 80%) pregnancy percentages at two years of age. There appears to be no reason to supplement EW heifers with true protein or with any supplement form (e.g. grain, protein meals) unless it seems likely that the target LW and BCS required for successful pregnancy will not be achieved without some form of supplement due to the low LW of the heifer at weaning. This becomes an economic rather than a biological decision.

### Longitudinal growth and IGF-1 concentration

The observed increases in LWG and measurements of growth rate of various skeletal parameters when feeding supplements were accompanied by changes in the concentration of hormones in the plasma as well as morphological changes of the growth plate. Supplemented heifers had a higher concentration of insulin, IGF-1 and T3 in the plasma and thicker PZ and HZ and larger terminal hypertrophic chondrocytes in bone. Similarly, Pando et al. [46] reported severe reductions in the concentration of circulating IGF-1 (reduced to about 20% of concentration in control animals) during caloric restrictions associated with a smaller epiphyseal growth plate in rats. Moreover, Wang et al. [47] demonstrated that IGF-1 null mice have a smaller HZ and terminal hypertrophic chondrocytes as well as slower bone elongation rate when compared to wild-type mice. Nutritional effects on endocrine and morphological parameters of the growth plate have been previously reported in rabbits [48], rodents [49–51] and cattle [26].

Longitudinal bone growth at the growth plate is regulated by a complex system of endocrine signals [52]. Many hormones can individually regulate the growth plate locally or in concert with other metabolic signals [53], although changes in the concentration of growth regulating hormones (e.g. GH and IGF-1) are not able to explain the observed changes in long bone growth rate observed over a lifetime [42]. For instance, the uncoupling of hormone concentration and bone elongation in the long term can be observed comparing the IGF-1 concentration between EW and NW across the different seasons of this experiment with the skeletal growth rate assessed by body measurements. The IGF-1 concentration was significantly higher in NW heifers in three out of the four sample collections (e.g. start of first dry season, early and end of first wet season) compared to EW, however the increase in HH was significantly greater in EW than NW across all these time points. This observation agrees with the previous assumption that growth plate maturation is the main factor setting limits to the rate of growth plate endochondral ossification.

### The effect of supplementation on the trabecular bone structure and bone biomarkers plasma concentrations

In humans and rodents, it is known that caloric restriction results in a decrease in bone formation and, occasionally, an increase in bone resorption [54, 55]. In order to detect physiological changes in bone turnover, circulating bone metabolism biomarkers can be measured as an indicator of these processes [56]. Bone-specific alkaline phosphatase concentration, as a marker of bone formation, showed a tendency to be higher (*P* = 0.056) in the plasma of supplemented heifers (i.e. S10) compared with unsupplemented (i.e. S0) heifers. In contrast, CTX-1, as a marker of bone resorption, was unaffected by supplementation. Supplementation led to a greater rate of HH and HW growth, therefore more bone was being mineralized to keep pace with the increased elongation rate which helps to explain the tendency for higher concentration of BALP in the plasma of these heifers despite similar trabecular bone structure between the two groups.

The concentration of bone metabolism biomarkers in plasma are normally higher in young compared with mature animals due to the faster growing skeleton which requires an accelerated process of bone mineralization and resorption [57, 58]. In addition, the variability in the concentration of bone biomarkers is greater in young compared with mature animals, which has been demonstrated for deoxypyridinoline [57] and BALP [58]. Taken together these results seem to indicate that when offered a high quality diet, fast growing young cattle have the potential to increase bone remodelling leading to a higher concentration of bone formation and resorption markers in the circulation. However, young cattle fed low quality diets have bone biomarker concentration similar to mature animals with low bone turnover activity, such as mature dry cows [59, 60].

### Long-term effect of post-weaning supplementation on frame size

In the present experiment compensatory growth in LW was observed over the first wet season, however there was no evidence of catch-up growth in the skeleton during this period. Interestingly, heifers with lower supplement intake during the post-weaning period had a significantly higher HHG (27 *vs*. 19 mm/100 days for S0 and S5; *P* < 0.05) and HWG (18 *vs*. 12 mm/100 days for S1 and S10; *P* < 0.05) during the second wet season, which indicates catch-up growth. This was observed approximately 1.5 years after the imposition of the supplement treatments and was contrary to our initial hypothesis. A similar response has been previously described by Fordyce and Chandra [61] as well as McLennan [62] when evaluating the effect of different supplementation strategies on growth paths of cattle raised in extensive grazing systems. These observations may indicate that the nutritional stress imposed at early weaning would not impair these heifers to achieve, at an older chronological age, a similar mature frame size of normally weaned cohorts. In addition, it suggests that catch-up growth may occur not only immediately after the suppression of the restrictive factor but at later stages of development leading to a longer growth phase due to a later closure of the epiphyseal growth plate.

It would be expected, however, that in the long-term the epiphyseal growth plate of nutritionally un-restricted heifers would undergo structural involution earlier than heifers which were subjected to restricted growth. In rats, a decline in proliferation rate and size of terminal hypertrophic chondrocytes during aging is also linked to a decrease in overall height of growth plate zones and number of chondrocytes per column [63, 64]. Children that experienced undernutrition early in life demonstrated increased height recovery when caloric intake was increased, although in some cases final height was not obtained until 25-years of age [65]. Incomplete catch-up has been reported by studies with rodents using a design of 40% food restriction for 10 days and *ad libitum* access to feed for 10 or 26 days during the re-alimentation period. However, by the end of the 26-day recovery the epiphyseal growth plate (includes resting, proliferative and hypertrophic zones) of rodents submitted to catch-up growth showed a smaller reduction in thickness when compared to control animals [46]. Therefore, the possibility that previously restricted rodents demonstrate a greater catch-up in skeletal size than that observed at 26 days or even complete catch-up (i.e. same body size as controls) at older stages of life should not be rejected.

In the present study, by the end of the first dry season heifers fed S10 treatment were 40 and 46 mm taller (assessed by HH) than unsupplemented (i.e. S0) heifers for NW and EW respectively. This difference was reduced to 27 and 28 mm by the end of the experimental period. Unfortunately, bone biopsies were not collected at the end of the experimental period to provide information on the histomorphometric parameters of the growth plate at this point in time. However, the growth path of HH as well as the growth rate in body dimensions during the second wet season suggests that none of the heifers had achieved their mature size by the end of the experimental period. For humans, an individual is classified as stunted when the height is more than two standard deviations below the mean height of the reference population based on the World Health Organization and the National Center for Health and Statistics [66]. Applying this same concept to the present study, the difference in HH between unsupplemented and supplemented heifers would need to be greater than 34 mm (average between EW and NW) to be classified as stunted. Therefore, the results presented in this work suggest that the absence of post-weaning protein supplementation to EW or NW replacement heifers does not have a long-term detrimental effect on mature size when compared to heifers offered S10 supplementation.

### Reproductive parameters

Increasing the percentage of weaned calves in a herd is a major challenge for tropical cattle breeding systems. Prolonged lactational anoestrus is a main factor limiting reproductive efficiency in cattle and a fairly common occurrence in northern Australia due to undernutrition [67, 68]. Early weaning, to manage lactation in cows, is a tool used to substantially increase pregnancy percentage, especially in harsher environments such as northern Australia, by reducing the nutrient demands of the cow [10, 13, 69]. The model developed in this work, which predicts the pregnancy percentage of two-year-old Brahman crossbred heifers based on PM.LW is a useful tool that has practical application. It suggests that two-year-old heifers need to have PM.LW of approximately 300 kg in October/November to achieve approximately 80% probability of pregnancy (67.1–90.3% for a 95% confidence interval), regardless of WW. Schatz and Hearnden [35] developed a similar model using data collected at the same location as this experiment. The authors proposed a target LW (curfew) of 253 kg measured in October/November would result in an 80% of pregnancy for two-year-old heifers. Similarly, working with two-year-old Brahman cross heifers Doogan et al. [36] et al. proposed approximately 270 kg as a target LW at the start of mating (January) in order to achieve a 80% confirmed pregnancy at the end of a 100-day mating period

## Conclusions

High occurrence of pregnancy (> 80%) in two-year-old Brahman crossbred heifers was achieved when mated at a LW of 300 kg or heavier, regardless of WW group. This may not be achieved without supplementation during the first dry season after weaning depending on the growth potential of heifers within the location and production system. Early weaned heifers offered a mineral urea lick block during the first dry season post-weaning did not show any indication that the lack of protein meal supplementation would lead to any long-term effect in terms of growth impediment in this environment (i.e. permanent stunting). However, EW heifers would require an additional year to achieve a target LW of 300 kg at the beginning of the mating season to achieve a pregnancy percentage of at least 80%.

## Supporting information

S1 File (XLSX)
